# Supplementary material for: Key pathological features characterize minimal change disease-like IgA nephropathy
Source: PLoS One. 2023 Jul 20;18(7):e0288384. doi: 10.1371/journal.pone.0288384 (PMC10358932; doi:10.1371/journal.pone.0288384)
Supplement: S1 Table — (PDF) [file pone.0288384.s001.pdf]

S1 Table. Individual renal biopsy pathology characteristics

Patient ID	Oxford classification	Foot Process effacement	Total Glomeruli examined in EM	Immunofluorescence stain pattern and intensity			
				IgA	IgG	IgM	C3
**1**	M1E0S0T1	Global	5	D,G,GR,M,++	-	-	D,G,GR,M,+
**2**	M0E0S0T0	Segmental	5	D.G,GR,M,++	-	-	D,G,GR,M,TR
**3**	M1E0S0T0	Segmental	7	D,G,GR,M,+++	-	-	D,G,GR,M,++
**4**	M0E0S0T0	Segmental	5	D,G,GR,M,+++	-	-	D,G,GR,M,++
**5**	M0E0S0T0	Segmental	1	D,G,GR,M,+++	-	-	D,G,GR,M,++
**6**	M1E0S0T0	Segmental	3	D,G,GR,M,++	D,G,GR,M,TR	D,G,GR,M,TR	D,G,GR,M,+
**7**	M1E0S0T0	Global	3	D,G,GR,M,++	-	D,G,GR,M,+	D,G,GR,M,,+
**8**	M1E0S0T0	Segmental	3	D,G,GR,M,+++	D,G,GR,M,+	-	D,G,GR,M,++
**9**	M1E0S0T0	Global	11	D,G,GR,M,+++	-	D,G,GR,M,+	D,G,GR,M,++
**10**	M0E0S0T0	Segmental	12	D,G,GR,M,+++	-	D,G,GR,M,+	D,G,GR,M,+
**11**	M0E0S0T0	Segmental	1	D,G,GR,M,++	-	D,G,GR,M,+	D,G,GR,M,TR
**12**	M1E0S0T1	Global	7	D,G,GR,M,+++	D,G,GR,M,+	D,G,GR,M,+	D,G,GR,M,+
**13**	M1E0S0T0	Global	1	D,G,GR,M,+++	-	D,G,GR,M,+	D,G,GR,M,++
**14**	M1E0S0T0	Global	2	D,G,GR,M,+++	-	-	D,G,GR,M,+
**15**	M1E0S0T0	Global	4	D,G,GR,M,+++	D,G,GR,M,+	D,G,GR,M,+	D,G,GR,M,+
**16**	M1E0S0T0	Global	1	D,G,GR,M,++	-	D,G,GR,M,+	D,G,GR,M,+
**17**	M0E0S0T0	Segmental	8	D,G,GR,M,++	-	D,G,GR,M,+	D,G,GR,M,+

**18**	M1E0S0T0	Segmental	7	D,G,GR,M,++	-	-	D,G,GR,M,+
**19**	M0E0S0T0	Global	7	D,G,GR,M, +++	D,G,GR,M,+	-	D,G,GR,M,+
**20**	M1E0S0T0	Segmental	5	D,G,GR,M,++	D,G,GR,M,+	D,G,GR,M,+	D,G,GR,M,TR
**21**	M1E0S0T0	Segmental	5	D,G,GR,M,+++	D,G,GR,M+	-	D,G,GR,M,++
**22**	M1E0S0T0	Segmental	3	D,G,GR,M,+++	-	D,G,GR,M,+	D,G,GR,M,++
**23**	M0E0S0T0	Segmental	3	D,G,GR,M,+	-	D,G,GR,M,TR	-
**24**	M1E1S0T0	Segmental	6	D,GR,GR,M,++	-	-	-
**25**	M1E0S0T1	Segmental	3	D,G,GR,M,+	-	D,G,GR,M,TR	TR
**26**	M0E0S0T0	Global	3	D,G,GR,M,++	-	-	D,G,GR,M,TR
**27**	M0E0S0T0	Segmental	3	D,G,GR,M,++	-	D,G,GR,M,+	D,G,GR,M,TR
**28**	M1E1S0T0	Segmental	12	D,G,GR,M,+++	-	D,G,GR,M,+	D,G,GR,M,++
**29**	M0E1S0T0	Segmental	9	D,G,GR,M,+++	-	D,G,GR,M,+	D,G,GR,M,++
**30**	M0E0S0T1	Segmental	4	D,G,GR,M,+++	-	D,G,GR,M,++	TR
**31**	M1E0S0T0	Segmental	6	D,G,GR,M,+++	D,G,GR,M,+	TR	D,G,GR,M,++
**32**	M1E0S0T1	Segmental	4	D,G,GR,M,+++	-	D,G,GR,M,+	D,G,GR,M,++
**33**	M0E0S0T0	Segmental	2	D,G,GR,M,++	-	-	-
**34**	M0E0S0T0	Segmental	4	D,G,GR,M,+++	-	-	D,G,GR,M,++
**35**	M1E0S0T0	Segmental	6	D,G,GR,M,+++	-	-	D,G,GR,M,TR
**36**	M0E0S0T0	Global	1	D,G,GR,M,+++	-	-	D,G,GR,M,++
**37**	M0E0S0T0	Global	7	D,G,GR,M,+++	D,G,GR,M,+	TR	D,G,GR,M,++
**38**	M0E0S0T0	Segmental	13	D,G,GR,M,+++	D,G,GR,M,+	-	D,G,GR,M,+
**39**	M0E0S0T0	Global	2	D,G,GR,M,+	-	-	D,G,GR,M,+
**40**	M0E0S0T0	Global	8	D,G,GR,M,+	-	-	D,G,GR,M,+

**41**	M0E0S0T0	Global	1	D,G,GR,M,+	-	D,G,GR,M,+	D,G,GR,M,TR
**42**	M0E0S0T0	Global	11	D,G,GR,M,++	D,G,GR,M,+	D,G,GR,M,+	-
**43**	M0E0S0T0	Global	7	D,G,GR,M,++	-	-	-
**44**	M0E0S0T0	Global	4	D,G,GR,M,++	D,S,GR,M,TR	D,S,GR,M,TR	D,G,GR,M,TR

Immunofluorescence stain pattern: Diffuse (D)/Focal(F); Global(G)/Segmental(S); Granular(GR)/Linear (L); Mesangial (M)/Capillary wall pattern(C); Immunofluorescence stain intensity: Trace(TR)/+/++/+++; EM: electron microscopy
