# Supplementary material for: Key pathological features characterize minimal change disease-like IgA nephropathy
Source: PLoS One. 2023 Jul 20;18(7):e0288384. doi: 10.1371/journal.pone.0288384 (PMC10358932; doi:10.1371/journal.pone.0288384)
Supplement: S2 Table — (PDF) [file pone.0288384.s002.pdf]

S2 Table. Individual IST and RAAS Blockade Regimen

Patient ID	Group	Initial IST	IST duration (months)	RAAS blockade
**1**	2	-	N/A	-
**2**	Excluded for UPCR<1g/g	prednisolone 30mg/day	52	-
**3**	Excluded for prior RAAS blockade	-	N/A	-
**4**	4	-	N/A	-
**5**	3	prednisolone 60mg/day	26	-
**6**	Excluded for lost follow-up	-	N/A	-
**7**	1	methylprednisolone 32mg/day<br>cyclophosphamide 100mg/day	4	-
**8**	3	prednisolone 30mg/day	60	-
**9**	2	-	N/A	-
**10**	4	-	N/A	-
**11**	4	-	N/A	-
**12**	1	methylprednisolone 32mg/day<br>cyclophosphamide 100mg/day	23	Irbesartan 150mg BID
**13**	2	-	N/A	-
**14**	1	methylprednisolone 32mg/day<br>cyclophosphamide 100mg/day	93	Losartan 50mg QD
**15**	Excluded for prior RAAS blockade	prednisolone 30mg/day<br>cyclophosphamide 100mg/day	6	-
**16**	Excluded for prior RAAS blockade	methylprednisolone 8mg/day	43	-
**17**	Excluded for UPCR<1g/g	prednisolone 15mg/day	82	-
**18**	4	-	N/A	Losartan 50mg QD
**19**	2	-	N/A	-
**20**	Excluded for lost follow-up	-	N/A	-
**21**	Excluded for lost follow-up	-	N/A	-

**22**	3	methylprednisolone 32mg/day	10	Losartan 50mg QD
**23**	Excluded for UPCR<1g/g	-	N/A	-
**24**	4	-	N/A	Telmisartan 80mg QD
**25**	4	-	N/A	-
**26**	1	methylprednisolone 40mg/day	2	-
**27**	Excluded for prior RAAS blockade	-	N/A	-
**28**	4	-	N/A	Losartan 50mg QD
**29**	3	prednisolone 25mg/day	28	-
**30**	Excluded for UPCR<1g/g	methylprednisolone 20mg/day	9	-
**31**	Excluded for prior RAAS blockade	-	N/A	-
**32**	Excluded for lost follow-up	-	N/A	-
**33**	Excluded for lost follow-up	-	N/A	-
**34**	4	-	N/A	Losartan 50mg QD
**35**	1	prednisolone 70mg/day	1	-
**36**	1	prednisolone 50mg/day	9	Losartan 50mg BID
**37**	4	-	N/A	-
**38**	3	prednisolone 20mg/day<br>mycophenolate mofetil 50mg/day	4	-
**39**	1	prednisolone 45mg/day	3	-
**40**	2	-	N/A	-
**41**	1	prednisolone 80mg/day	18	-
**42**	1	prednisolone 50mg/day	3	-
**43**	1	methylprednisolone 16mg/day<br>cyclophosphamide 50mg/day	6	-
**44**	1	methylprednisolone 8mg/day<br>cyclophosphamide100mg/day	7	Losartan 50mg QD

IST: immunosuppressant therapy; RAAS: renin angiotensin aldosterone system; N/A: not applicable; QD: once daily; BID: twice daily
