# Supplementary material for: Key pathological features characterize minimal change disease-like IgA nephropathy
Source: PLoS One. 2023 Jul 20;18(7):e0288384. doi: 10.1371/journal.pone.0288384 (PMC10358932; doi:10.1371/journal.pone.0288384)
Supplement: S3 Table — (PDF) [file pone.0288384.s003.pdf]

S3 Table. Individual clinical characteristics according to steroid response

Patient ID	Group	response	Blood pressure (Systolic/Diastolic) mmHg	BMI kg/m2	IST duration (months)	RAAS blockade
**5**	3	NR	105/66	26.2	26	-
**7**	1	CR	121/84	29.6	4	-
**8**	3	CR	135/64	20.9	60	-
**12**	1	NR	136/83	25.4	23	Irbesartan 150mg BID
**14**	1	CR	119/71	23	93	Losartan 50mg QD
**22**	3	PR	139/77	18.2	10	Losartan 50mg QD
**26**	1	CR	125/75	27.4	2	-
**29**	3	PR	126/88	28.7	28	-
**35**	1	CR	134/84	26.4	1	-
**36**	1	PR	143/82	21.9	9	Losartan 50mg BID
**38**	3	NR	167/76	22.2	4	-
**39**	1	CR	121/67	33.3	3	-
**41**	1	CR	112/69	27.4	18	-
**42**	1	CR	121/81	27.8	3	-
**43**	1	CR	177/88	26.9	6	-
**44**	1	CR	101/54	35.2	7	Losartan 50mg QD

NR: no response; PR: partial remission ; CR: complete remission; IST: immunosuppressant therapy; RAAS: renin angiotensin aldosterone system; BMI: body mass index; QD: once daily; BID: twice daily
