# Supplementary material for: Key pathological features characterize minimal change disease-like IgA nephropathy
Source: PLoS One. 2023 Jul 20;18(7):e0288384. doi: 10.1371/journal.pone.0288384 (PMC10358932; doi:10.1371/journal.pone.0288384)
Supplement: S1 File — (PDF) [file pone.0288384.s006.pdf]

Staining protocol

The biopsy specimens were obtained through percutaneous ultrasound-guided kidney biopsy and divided into three parts for light microscopy, immunofluorescence, and electron microscopy. For light microscopic examination, the kidney tissue was fixed in 10% neutral buffered formalin, and processed through dehydration in alcohol, clearing in xylene and infiltration with paraffin wax. The paraffin-embedded tissue block was prepared and cut into 3 μm sections by a microtome. The slides were deparaffinized and routinely stained with hematoxylin and eosin using the Leica ST5010 Autostainer XL staining system (Leica Microsystems, Germany). The histochemical stains for Masson trichrome, periodic acid-Schiff, and Jones' methenamine silver were all performed on Agilent Dako Artisan Link Pro slide stainer (Agilent, USA) according to manufacturer's protocols. The tissue core for immunofluorescence was kept in fresh and embedded in optimal cutting temperature compound (OCT) compound for frozen sectioning on a cryostat microtome. The 5μm sections were cut and washed in wash buffer (dilution 1:10, code S3006, Dako, Agilent, USA). The slides were incubated with polyclonal fluorescein isothiocyanate-conjugated rabbit anti-human antibodies to IgG (dilution 1:20, code F0202, Dako), IgA (dilution 1:20, code F0204, Dako), IgM (dilution 1:20, code F0203, Dako), C1q (dilution 1:20, code F0254, Dako), C3 (dilution 1:20, code F0201, Dako), C4 (dilution 1:20, code F0169, Dako), kappa (dilution 1:40, code F0198, Dako), and lambda light chains (dilution 1:40, code F0199, Dako) for 30 minutes at room temperature. The slides were washed in wash buffer, mounted in mounting medium (code S3023, Dako) and viewed under Olympus BX63 fluorescence microscope (Olympus, Japan). For electron microscopy, specimens were fixed in 2.5% glutaraldehyde in phosphate buffer, postfixed with 1% osmium tetroxide (OsO4) in phosphate buffer and further dehydrated with a graded series of ethanol, and embedded in Spurr’s EPON. Ultrathin sections with a thickness of 90 nm were cut and contrasted with uranyl acetate and lead citrate. The ultrastructural findings were examined and photographed on a JEM-1400 electron microscope (JEOL, Japan).
