# Supplementary material for: Key pathological features characterize minimal change disease-like IgA nephropathy
Source: PLoS One. 2023 Jul 20;18(7):e0288384. doi: 10.1371/journal.pone.0288384 (PMC10358932; doi:10.1371/journal.pone.0288384)
Supplement: Attachment — Submitted filename: Response to Reviewers (PONE-D-22-24589R1).docx [file pone.0288384.s007.docx]

**Point-by-point responses to the editor and reviewers (PONE-D-22-24589R1)**

i) **need to acknowledge the limitations related to the small sample size of the single groups and the differing treatment protocols;**

**=> Responses:** Thanks for your valuable comment. We added small sample size, and variance of regimen in the part of limitation in lines 313-315 of the revised manuscript with track changes, as follows.
Third, sample size was small, owing to low prevalence of the IgAN with MCD. Lastly, different IST regimens were implemented in the treatment groups.

ii) **need further discussion of the data;**
    **=> Responses:** Thanks for your suggestion. We added relevant discussion of Oxford classification in lines 228-240 as follows.
    Absence of endocapillary hypercellularity (E = 0) was not required in our cohort. Though all of the patient with global FPE happened to be classified as E = 0, the proportion had no significant difference to that of the segmental group. Additionally, one drawback of endocapillary hypercellularity scoring is that its reproducibility was poorer than segmental sclerosis scoring. Counting macrophage numbers in CD68-stained section of glomerulus may be an appropriate alternative for endocapillary hypercellularity scoring.
    Tubulointerstitial fibrosis (T = 1 or 2) is frequently associated with hypertension in adults, particularly in the elderly, and does not necessarily exclude MCD. So it was not listed as an exclusion criteria in our study. In a multi-center retrospective cohort, patients with pathological change T1 were found to be more resistant to steroid. In our cohort, three patients were resistant to steroid in treatment group and two of them were pathologically classified as T = 1. By contrast, none of the steroid-responsive IgAN patients were pathologically classified as T = 1 or 2.
iii) **need to reorganize the manuscript to improve legibility and easy understanding of the issues presented;**
     **=> Responses:** Thanks for your comment. We made some improvement to our article, listed as points below:
     1. Descriptions about IST regimen, and group stratification were added so as to better understanding the article in lines 116-120 of the revised manuscript with track changes, as follows.
        The IST regimen was either steroid alone, steroid in combination with cyclophosphamide, or steroid in combination with mycophenolate mofetil. Global FPE with IST, global FPE without IST, segmental FPE with IST, and segmental FPE without IST were designated as group 1, group 2, group 3, and group 4 respectively.
     2. We reorganized the paragraph discussing immunosuppressive therapy in MCD-like IgAN in lines 248-269 of the revised manuscript with track changes, as follows.
        KDIGO guidance recommends that IgAN with MCD-like features should be treated as MCD [1]. However, the recommendation lack valid evidence because of the rare population, and the related studies enrolled IgAN patients with nephrotic syndrome rather than IgAN with MCD-like pathological features [2-4]. In our study, IST in MCD-like IgAN patients attenuated the eGFR decline rate and had better renal outcomes than the non-IST-treated group. These patients mostly achieved CR, and the time to IST was within 3 months. Only one patient classified as MCD-like IgAN was defined as NR in treatment response. Whether the resistant response to first-line glucocorticoid treatment in MCD-like IgAN patients suggest poor renal outcomes needs further investigation. In condition of MCD, a substantial portion of patients progressed to ESKD if unresponsive to glucocorticoid treatment.
     3. We deleted the paragraph discussing immunosuppressive therapy in non MCD-like IgAN patients for simplicity in lines 280-284 of the revised manuscript with track changes.
iv) need to address the remaining detailed comments of Reviewer 1 (concerning abstract, introduction, figures, tables, and discussion), all relevant to definitely improve the presentation and clarity of the manuscript.
    **=> Responses:**
    Thanks for your comment. We addressed comments of Reviewer 1 and the details are presented in the following section.

Reviewer #1

**Abstract:**

**Line 5: We aimed to identify the key pathological features of MCD-like IgAN.**

**- that was the reason for the question, if your work would support, that also E0 could be a key pathological feature as 100% in Global FPE are E0 (but also 88.5 in the segmental FPE Group – so the answer is probably no).**

**=> Responses:** Thanks for your comment. We added relevant descriptions in lines 228-230 of the revised manuscript with track changes, as follows.

Absence of endocapillary hypercellularity (E = 0) was not required in our cohort. Though all of the patient with global FPE happened to be classified as E = 0, the proportion had no significant difference to that of the segmental group.

**Introduction:**

**Line 25: I would suggest you the following: The frequent clinical manifestations are asymptomatic hematuria and proteinuria of differing degrees. I would also strongly suggest to give more background information on published work concerning MCD IgAN.**

**=> Responses:** Thanks for your comment. We added information about MCD-like IgAN in lines 25-29 of the revised manuscript with track changes, as follows.

Nephrotic syndrome occurs in 5% of IgAN patients[5]. IgAN with nephrotic syndrome is considered to be a variant form of IgAN and can have electron microscopic features resembling minimal change disease (MCD). The treatment strategy in this MCD-like variant form is in accordance with MCD rather than IgAN.

**Line 58- Line 65: Confusing explanation and grammar. I would suggest not to add this to the manuscript. (See also comment to line 5). The question was rather, if tubulointerstitial fibrosis interferes with proteinuria which could have an impact on your clinical parameters (CR, PR and NR).**

**=> Responses:** Thanks for your suggestions. This paragraph was meant to explain why E1 and T1 were not implemented as exclusion criteria. We revised and putted this paragraph into discussion section in lines 228-240 of the revised manuscript with track changes, as follows.

Absence of endocapillary hypercellularity(E = 0) was not required in our cohort. Though all of the patient with global FPE happened to be classified as E = 0, the proportion had no significant difference to that of the segmental group. Additionally, one drawback of endocapillary hypercellularity scoring is that its reproducibility was poorer than segmental sclerosis scoring[6]. Counting macrophage numbers in CD68-stained section of glomerulus may be an appropriate alternative for endocapillary hypercellularity scoring[7].

Tubulointerstitial fibrosis (T = 1 or 2) is frequently associated with hypertension in adults, particularly in the elderly, and does not necessarily exclude MCD[8]. So it was not listed as an exclusion criteria in our study. In a multi-center retrospective cohort, patients with pathological change T1 were found to be more resistant to steroid[9]. In our cohort, three patients were resistant to steroid in treatment group and two of them were pathologically classified as T = 1. By contrast, none of the steroid-responsive IgAN patients were pathologically classified as T = 1 or 2.

**Line 73-113:**

**Describe in this paragraph the immunosuppression used for the patient groups to facilitate the understanding. IST is not only Steroids but often also CYC in your population.**

**=> Responses:** Thanks for your comment. We added relevant descriptions as below in lines 116-118 of the revised manuscript with track changes:

The IST regimen was either steroid alone, steroid in combination with cyclophosphamide, or steroid in combination with mycophenolate mofetil.

**Revised Figure 2: The groups that are defined in Figure 2 need to be described and defined in the material and methods section. Why did you exclude 4 patients with UPCR <1 g from the segmental FPE with IST group?**

**=> Responses:** Thanks for your comment.

Global FPE with IST, global FPE without IST, segmental FPE with IST, and segmental FPE without IST were designated as group 1, group 2, group 3, and group 4 respectively. We added above descriptions in lines 118-120 of the revised manuscript with track changes.

According to KDIGO guideline, glucocorticoid treatment is not considered in classical IgAN patient with UPCR < 1g/day after maximal supportive treatment. Therefore, patients with UPCR < 1g/day in group 3 (segmental FPE with IST) were excluded.

**Line 78: The stainings are mentioned but your staining protocols are still missing. I suggest to add these protocols to the manuscript**

**=> Responses:** Thanks for your comment. The staining protocol was added in supplementary information.

**Line 107: better nephrotic range proteinuria**

**=> Responses:** Thanks for your suggestions. We revised the paragraph in lines 109-110 of the revised manuscript with track changes, as follows.

Nephrotic range proteinuria was defined as UPCR >3.5 g/g. Nephrotic syndrome was defined as nephrotic range proteinuria, serum albumin < 2.5 g/dL, and presence of edema.

**Line 116: It is always desirable to follow the KDIGO guidelines but maybe not necessary to cite here, when you define your own remission criteria which are (to my best knowledge) not displayed 1:1 in the KDIGO Guidelines IgAN section.**

**=> Responses:** Thank you so much for your suggestion. We modified this sentence in lines 123-126 of the revised manuscript with track changes, as follows.

The response to IST was defined as complete remission (CR) if UPCR < 0.3 g/g after treatment, partial remission (PR) if proteinuria reduction > 50% but UPCR > 0.3 g/g, and no response (NR) if proteinuria reduction < 50%.

**Line 119: Please add: At which timepoint you decided if CR; NR oder PR were achieved. At the end of IST?**

**=> Responses:** Thanks for your comment.

The follow-up UPCR was assessed approximately 3 months after initiation of IST. We added above statement in lines 126-127 of the revised manuscript with track changes.

**Line 166: better nephrotic range proteinuria.**

**=> Responses:** Thanks for your suggestion. We added relevant sentence in lines 172-173 of the revised manuscript with track changes, as follows.

Fourteen (31.8%) of the enrolled patients had nephrotic range proteinuria.

**Table 4: Clinical Characteristics: Add N numbers of the groups. The legend contains CR, PR and NR which are until now not displayed in the table.**

**Table S3: Spironolactone is no RAS Inhibitor.**

**=> Responses:** Thanks for your valuable comment. Spironolactone was only prescribed in patient no. 7. We modified RAAS blockade use of patient no. 7 in data S5. We deleted spironolactone in the RAAS blockade column in Table S2 and Table S3. N numbers of each groups were added in Table 4 and RAAS blockade proportion was recalculated. CR, PR, an NR were removed from the figure legend since these words were placed erroneously.

**Figure 3: Where are the figure legends? P- value described in the text not in the figure.**

**The figures are not in the right order.**

**=> Responses:** Thanks for your comment. We added relevant description in figure legend as below: Complete remission was achieved in 81.8% of the global FPE group and 20% in the segmental FPE group (p = 0.018).

We checked the figure order, and it was correct.

**Line 195-199 Information found in table 3? Please mention in the text. Add group numbers from the text to the table 3. I.e. Group 1=Global FPE(IST+) for better and quicker understanding.**

**=> Responses:** Thanks for your comment. We added group numbers in the table 3 and relevant descriptions in the text in lines 204-205 of the revised manuscript with track changes, as follows.

eGFR change rate was compared between groups 1 and 2, groups 3 and 4, groups 1 and 3, and groups 2 and 4, as shown in Table 3.

**Discussion:**

**Line 218-224: What is your conclusion drawn from these informations?**

**=> Responses:** Thanks for your comment. This paragraph is meant to compare the prevalence of nephrotic syndrome in IgAN in our cohort with previous studies and it was similar to our study.

**Line 225: Not clear, you mean that the KDIGO guidelines recommend treatment with steroids?**

**=> Responses:** Thanks for your comment. We modified the sentence in lines 248-249 of the revised manuscript with track changes, as follows.

KDIGO guidance recommends steroid treatment in IgAN with MCD-like features.

**Line 243-255: The discussion on generally treating IgAN with steroids or not does rather not apply to your study- The studies cited here did not focus on MCD like IgAN. I would suggest not to discuss that.**

**=> Responses:** Thanks for your comment. The two studies cited in this paragraph found that immunosuppression therapy ameliorate eGFR decline rate in IgAN patients with no MCD-like features. In our cohort, patients in group 3 (segmental FPE with IST) appeared to have slower eGFR decline rate than group 4(segmental FPE without IST), though it had no significant statistical difference. For simplicity, we subtracted the paragraph in lines 280-284 of the revised manuscript with track changes.

**Line 285 maybe you could explain the importance of the former sentences for your study?**

**=> Responses:** Thanks for your comment. We considered that the MCD-like features in IgAN was more likely to be coexistence of MCD rather than podocytopathic variant of IgAN, as discussed in lines 285-309 of the revised manuscript with track changes.

**Line 293: Limitations: I would suggest to add the small group sizes, the differences in IST treatment in dose and drugs (Group 3 did never receive CYC) and that finally an negative effect of higher blood pressure in group 3 cannot be excluded.**

**=> Responses:** Thanks for your comment. We added small sample size, and variance of regimen in the part of limitation in lines 313-315 of the revised manuscript with track changes, as below.

Third, sample size was small, owing to low prevalence of the IgAN with MCD. Lastly, different IST regimens were implemented in the treatment groups.

**I would suggest not mention that you do not have the possibility to measure gd-IgA as a limitation. It would have been interesting for a discussion about IgAN activity in patients with simultaneous MCD features but is not strictly necessary.**

**=> Responses:** Thank you so much for your suggestion. We deleted this description.

**Concerning answer 6) and the ESRD patients. It is quite surprising, that with these Creatinine values at diagnosis the patients had still an S0. Can you comment on this?**

**=> Responses:** Thanks for your comment. In IgAN patients with eGFR <30ml/min/1.73m^2^, approximately 8% was pathologically classified as S0[10]. It is possible for a IgAN patient with advanced renal impairment to be classified as S0.

Reviewer #2: (No Response)

References:

1.	KDIGO 2021 Clinical Practice Guideline for the Management of Glomerular Diseases. Kidney Int. 2021;100(4s):S1-s276. Epub 2021/09/25. doi: 10.1016/j.kint.2021.05.021. PubMed PMID: 34556256.

2.	Pattrapornpisut P, Avila-Casado C, Reich HN. IgA Nephropathy: Core Curriculum 2021. Am J Kidney Dis. 2021;78(3):429-41. Epub 2021/07/13. doi: 10.1053/j.ajkd.2021.01.024. PubMed PMID: 34247883.

3.	Lai KN, Lai FM, Ho CP, Chan KW. Corticosteroid therapy in IgA nephropathy with nephrotic syndrome: a long-term controlled trial. Clin Nephrol. 1986;26(4):174-80. Epub 1986/10/01. PubMed PMID: 3536231.

4.	Kim SM, Moon KC, Oh KH, Joo KW, Kim YS, Ahn C, et al. Clinicopathologic characteristics of IgA nephropathy with steroid-responsive nephrotic syndrome. J Korean Med Sci. 2009;24 Suppl(Suppl 1):S44-9. Epub 2009/02/12. doi: 10.3346/jkms.2009.24.S1.S44. PubMed PMID: 19194561; PubMed Central PMCID: PMCPMC2633194.

5.	Barratt J, Feehally J. IgA nephropathy. J Am Soc Nephrol. 2005;16(7):2088-97. Epub 2005/06/03. doi: 10.1681/asn.2005020134. PubMed PMID: 15930092.

6.	Roberts IS, Cook HT, Troyanov S, Alpers CE, Amore A, Barratt J, et al. The Oxford classification of IgA nephropathy: pathology definitions, correlations, and reproducibility. Kidney Int. 2009;76(5):546-56. Epub 2009/07/03. doi: 10.1038/ki.2009.168. PubMed PMID: 19571790.

7.	Soares MF, Genitsch V, Chakera A, Smith A, MacEwen C, Bellur SS, et al. Relationship between renal CD68(+) infiltrates and the Oxford Classification of IgA nephropathy. Histopathology. 2019;74(4):629-37. Epub 2018/10/12. doi: 10.1111/his.13768. PubMed PMID: 30303541.

8.	Vivarelli M, Massella L, Ruggiero B, Emma F. Minimal Change Disease. Clin J Am Soc Nephrol. 2017;12(2):332-45. Epub 2016/12/13. doi: 10.2215/cjn.05000516. PubMed PMID: 27940460; PubMed Central PMCID: PMCPMC5293332.

9.	Yang P, Chen X, Zeng L, Hao H, Xu G. The response of the Oxford classification to steroid in IgA nephropathy: a systematic review and meta-analysis. Oncotarget. 2017;8(35):59748-56. Epub 2017/09/25. doi: 10.18632/oncotarget.19574. PubMed PMID: 28938678; PubMed Central PMCID: PMCPMC5601774.

10.	Coppo R, Troyanov S, Bellur S, Cattran D, Cook HT, Feehally J, et al. Validation of the Oxford classification of IgA nephropathy in cohorts with different presentations and treatments. Kidney Int. 2014;86(4):828-36. Epub 2014/04/04. doi: 10.1038/ki.2014.63. PubMed PMID: 24694989; PubMed Central PMCID: PMCPMC4184028.
